# Measurement of active shoulder proprioception: dedicated system and device

**DOI:** 10.1007/s00590-012-0950-y

**Published:** 2012-02-04

**Authors:** Przemyslaw Lubiatowski, Piotr Ogrodowicz, Marcin Wojtaszek, Ryszard Kaniewski, Jakub Stefaniak, Witold Dudziński, Leszek Romanowski

**Affiliations:** Department of Orthopedics, Traumatology and Hand Surgery, University of Medical Sciences in Poznan, Ul. 28 Czerwca 1956r 135/147, 62-545 Poznan, Poland

**Keywords:** Shoulder, Instability, Proprioception, Joint position sense, Angle reproduction

## Abstract

Proprioception is an essential part of shoulder stability and neuromuscular control. The purpose of the study was the development of a precise system of shoulder proprioception assessment in the active mode (Propriometr). For that purpose, devices such as the electronic goniometer and computer software had been designed. A pilot study was carried out on a control group of 27 healthy subjects, the average age being 23.8 (22–29) in order to test the system. The result of the assessment was the finding of the error of active reproduction of the joint position (EARJP). EARJP was assessed for flexion, abduction, external and internal rotation. For every motion, reference positions were used at three different angles. The results showed EARJP to range in 3–6.1°. The proprioception evaluation system (propriometr) allows a precise measurement of active joint position sense. The designed system can be used to assess proprioception in both shoulder injuries and treatment. In addition, all achieved results of normal shoulders may serve as reference to be compared with the results of forthcoming studies.

## Introduction

Shoulder stability depends on both passive (bony structures, capsule and ligaments) and active (muscles) stabilizers. Stability at rest is provided by negative pressure that is created by corresponding surfaces, a watertight capsule and joint fluid. While being in motion, the joint retains its stability by balancing muscle action and, by capsular and ligamentous restrains in extreme motion. The stabilization mechanism is controlled by the central nervous system [1–9]. Static and dynamic functions of joint stabilizers are integrated by the mechanism of proprioception.

Articular proprioception is defined as a specialized sensory function that includes the sensation of movement (kinaesthesia) and the joint position [5, 7, 10, 11]. This kind of neuromuscular control may become dysfunctional when the nervous reflex is disrupted. An injury to articular structures containing mechanoreceptors affects proper signalling to the central nervous system. On the other hand, however, an abnormal function of damage to the upper levels of the nervous system may affect neuromuscular control and, as a result, lead to an increased risk of injury.

The main objective of the research was to develop a method for assessing the proprioception of the glenohumeral joint, while being in motion, with the use of exact measurements and precise diagnosis.

## Materials and methodology

The scientific work had been divided into two phases. The first phase focused on the creation of a measuring device design and the development of the examination methodology. The second phase, however, focused on the introduction of pilot tests in order to measure the proprioception of a normal shoulder. This was granted by the State Committee for Scientific Research.

### Propriometer

The first phase of the scientific work required a device for measuring the glenohumeral joints’ proprioception, namely—the Propriometer. The construction works had been carried out in cooperation with the Progress Company (Ostrów Wielkopolski, Poland).

**Fig. 1:**
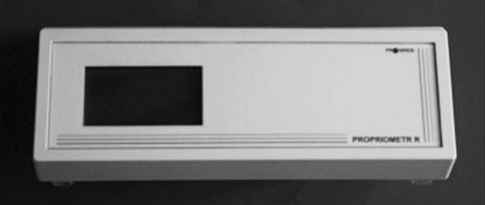
PC panel of Propriometer
- X-panel—a module of the uniaxial transducer of an arm’s position (Fig. 2), which is used in the analyses of abduction, flexion as well as external and internal rotation. The transducer measures the deviation of the transducers’ X-axis position, relatively to the direction of the Earth’s magnetic field lines. When being in the process of measurement, the Z-axis must be perpendicular to the direction of the field lines. The Z-axis direction, relativity to the meridians, has no effect on the results. The module is mounted to the patients’ arm with two straps. The X-panel contains the CMOS accelerometer (MEMSIC). This transmitter does not contain any mechanical components and its function is based on the heat flow phenomenon. The inertia mass is composed of a sphere of heated gas. Multiple, symmetrically placed, microthermal junctions, measure gas mixing, caused by the change of the sensor positioning relative to the Earth’s magnetic field.

The Propriometer is an electric goniometer that allows continuous evaluation of the deviation angle with the accuracy of 0.1°. The devise operates under both direct and PC control, and the whole set includes the following components:

- PC panel—groups all the elements of the measurement set with the PC (Fig. 1). It is additionally equipped with a display unit that presents the exact value of deviation angles.

**Fig. 2:**
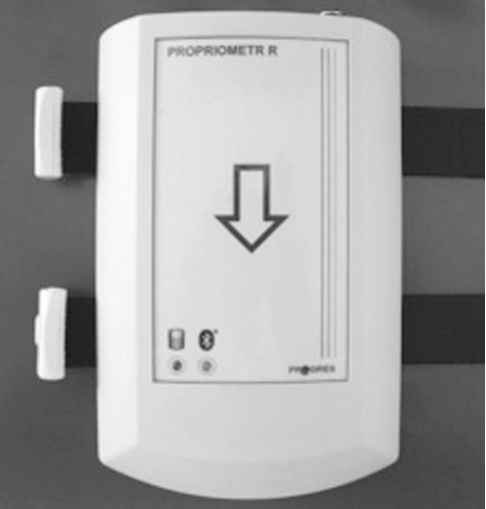
X-panel of Propriometer—module of uniaxial transducer of the arm’s position
- Remote control—used to confirm a joint position while the transducer is fixed to the arm. The general principle of the operation is based on the measurement of angular deviation of the transmitter with respect to the direction of the Earth’s magnetic field.
- Software and PC—the computer software had been specifically and exclusively designed for the purpose of device and measurement control (Fig. 3). The main role of the software is to control the values and direction of arm deviation, record and archive the patients’ data, together with the results of repeated measurements.

**Fig. 3:**
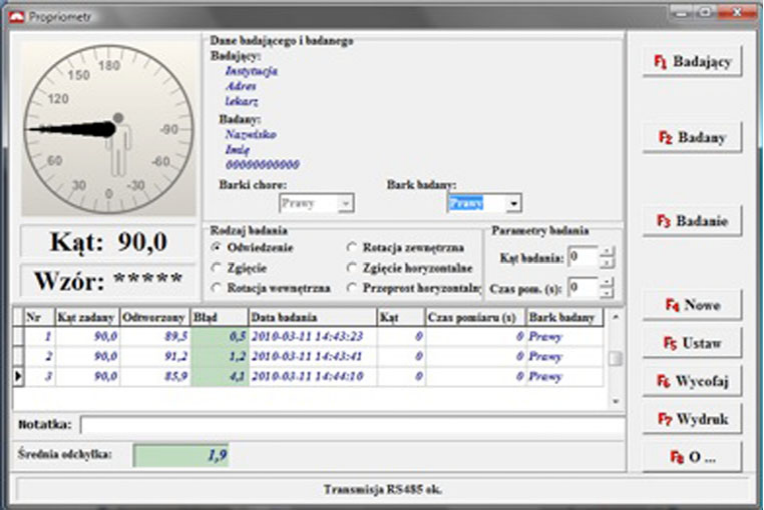
Screenshot depicting software controlling the Propriometer

It is possible to select the following directions of shoulder movement: flexion, abduction and, external and internal rotation. All patient data are stored in a database. Following the measurement procedure, a report of the examination can be printed, and the results can additionally be exported from the database to a spreadsheet program. All the results of a particular patient can be exported for additional database calculation purposes.

The Propriometer meets all standards that are required for medical devices. Power is supplied by safe 12 V. The PC panel has a separate galvanic voltage, in order to separate the computer from other elements. A precise manual has been attached to the device.

### Evaluation of shoulder proprioception by means of Propriometer

In the second phase of the scientific work, methodology of examining shoulder proprioception had been elaborated. For that purpose an active reproduction of the joint position (ARJP) was analysed. ARJP is based on a patients’ ability to reproduce a demonstrated reference angle by actively positioning the arm.

The final result of the research was the finding of an error connected with the active reproduction of the joint position (EARJP). EARJP is expressed in degrees, just as the difference between the reference angle and the angle reproduced by the patient. For further analysis, the absolute value of difference had been used.

### Test preparation

**Fig. 4:**
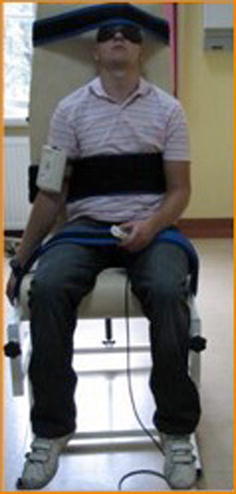
Proprioception examination set-up: examinee’s position for flexion and abduction position with assembled device

The X-panel was placed on the patients’ arm or forearm, depending on the direction of motion. The patient was instructed how to operate the device and what the methodology of the examination was. The study was performed in a room that provided a quiet environment to assure proper concentration. Covering the patient’s eyes eliminated vision. The task of the patient was to reproduce the demonstrated joint position as closely as possible (Fig. 4).

### Methodology of proprioception evaluation

The evaluation process started by demonstrating the reference angle of the patient’s arm to a desired position (active assisted motion). The position was to be held actively by the patient and then was confirmed and recorded by pressing the button of the remote control, being held in the opposite hand. Then, the arm returned to a neutral position and the examinee attempted to actively reproduce the reference position. Once the position had been achieved, the examinee had confirmed it by pressing the button on the remote control once more. The ultimate results of reference and reproduced angles had been recorded and added to the database.

**Fig. 5:**
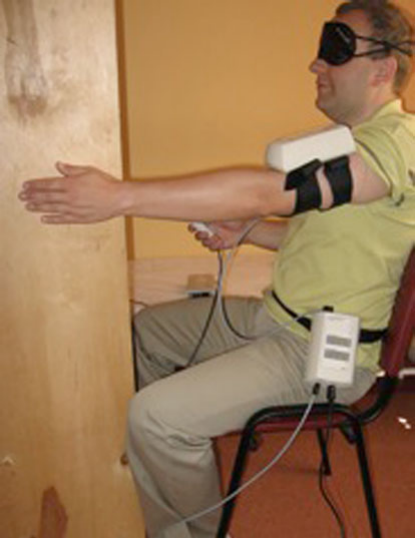
Evaluation of dynamic shoulder proprioception in flexion

The examination had been conducted in four directions of the motion: abduction, flexion and internal and external rotation. The reference angles for flexion and abduction had been suggested to be 60°, 90° and 120° (Fig. 5). For internal and internal rotation positions: 30°, 45° and 60° had been selected. Three repetitions had been performed for each direction of motion and reference position. The final result was obtained by calculating the absolute values of all three measurements.

**Fig. 6:**
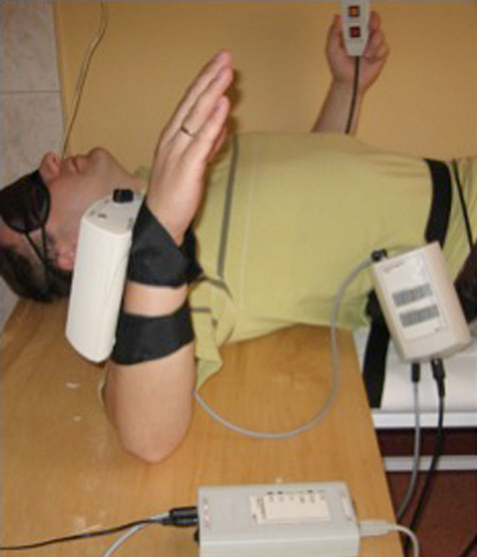
Evaluation of dynamic shoulder proprioception in rotation

Tests for both limbs are always performed. The final report that was obtained containing 24 EARJP results was based on 72 measurements. The examination of flexion and abduction had been performed on a person in a sitting position. The X-panel was placed on the tested arm. The flexion movements were examined in a sagittal plane, whereas abduction- in the plane of scapula. Both movements were performed in a natural rotation of the arm and began from a neutral arm position. The rotational movements were tested in supine position (Fig. 6). The X-panel was placed on the forearm of the tested site. The starting position was the abduction of the arm to 90° in neutral rotation.

### Material

The pilot tests had been performed on 27 healthy subjects, recruited from medical university students. The study had been granted proper ethical committee approval. The average age was 23.8 (21–29). There were 10 women and 17 men. All examined subjects had healthy, normal shoulders. There were no athletes in the group. The inclusion criteria included the following:

- age below 30
- no complaints in both glenohumeral joints
- no injuries and surgeries around the glenohumeral joint
- no neurological disorders
- correct function of the glenohumeral joint in basic clinical tests:
  - The range of motion,
  - The muscle test,
  - Impingement tests,
  - The instability tests.

We have used STAT PLUS for Mac 2009 for statistical analysis. Distribution of data was tested first and it proved to be normal. Then, ANOVA tests were used for further analysis.

## Results

Throughout the period of the study the device worked properly, without any failures. No safety problems had been recorded while testing the proprioception. There were no side effects or complaints. The assessment of the proprioception was not physically demanding, however, it did require a high level of concentration. It took one patient approximately 25–40 min to perform the proprioception assessment.

The results of EARJP have been presented in the Tables 1 and 2.

**Table 1:** Results of average EARJP for abduction and flexion

Movement	Reference position		
	60°	90°	120°
Abduction	5.1 ± 2.7	4.3 ± 1.9	4.2 ± 2.0
Flexion	6.1 ± 2.6	3.6 ± 1.9	3.7 ± 2.2

**Table 2:** Results of average EARJP for internal and external rotation

Movement	Reference position		
	30°	45°	60°
External rotation	3.4 ± 1.3	3.0 ± 1.0	3.0 ± 1.3
Internal rotation	3.9 ± 1.7	3.2 ± 1.4	3.0 ± 1.5

## Discussion

Proprioception is a specialized and complex sensory function; therefore, we may find a limited number of publications in the area [1, 4, 5, 11, 16, 24]. However, shoulder and knee instability has caused a vast increase of interest in the topic. The inspiration for these studies had been the finding of mechanoreceptors in the joint structures [1, 6, 12]. These structures are quite often subject to injuries and may lead to joint instability. The instability problem is deeper than just the damage to ligaments, labrum or tendons. It may also have an effect on neuromuscular control [1, 5, 7, 13, 14].

Abnormal proprioception may be a primary issue for some patients, causing it to be predisposed to injury. Therefore, this study was focused on the development of a method for shoulder proprioception evaluation represented by joint position sense (JPS).

JPS is the most common way of shoulder proprioception evaluation. It can be analysed in both passive and active motion. The main idea is to measure the error to reproduce the desired position of the joint [1, 5, 15, 16]. In this project, an active movement was chosen as the method of assessment, as the testing of active movement allows for the assessment of both afferent (sensory) and efferent components (nervous reflex; muscles) [17]. The studies of proprioception that particularly affect dynamic measurements of the glenohumeral joint are not numerous [17–20].

Techniques used in tests of existing publications differ from each other. In some works, the study had been based only on the rotational movements, while in other studies flexion and abduction had also been used. In one study, the final result was not EARJP but the angular position of movement. In another similar study, the final result was the distance in millimetres rather than the value of the angle.

In our methodology, however, we have relied on all the basic movements of the shoulder joint. The position of abduction and external rotation (ABER) is typical for the provocation of instability syndromes. The position of flexion and abduction influence the basic functional movement of the glenohumeral joint, which is used in most activities of daily life, such as reaching something from a shelf or lifting objects.

It is crucial to understand that the positions of the deviation are as important as the directions of movement. For each of the movements, three positions have always been studied: low deviation (60° of flexion and abduction; 30° of rotation), the medium deviation in the middle of the motions’ range (90° of flexion and abduction; 45° of rotation) and the high deviation (120° of flexion and abduction; 60° of rotation). The maximal deviations had not been used to avoid unwanted discomfort or situations when the patient could not perform such movement. The position of the joint can have a stimulating effect on mechanoreceptors. In theory, increased abduction or rotation causes more tension on the ligaments and capsule, and also evokes the pressure on the supraspinatus tendon against the acromion [22]. The way of demonstrating the angle of the reference position [21] may be also an important issue. In our study, the angle had been demonstrated by active assisted motion, which allows one to perform a test in the most repeatable way [1].

Most scientific work does not indicate the accuracy of digital measuring devices. The accuracy of analogue equipment is 1° [19]. Based on the literature, different measuring devices had been used for the position evaluation. Examples include electronic goniometers [1], analogue inclinometers [19], systems of cameras and markers [17, 18] and isokinetic dynamometers [23]. We also used an electronic goniometer with high measuring accuracy (0.1°). In order to correctly examine the JPS and kinaesthesia, it was necessary to use sensitive equipment. The accuracy of the test angle was supposed to be 0.1°. Differences of the angle in disorders of proprioception that were considered as significant, averaged around 0.6°–0.8° [7]. Devices with such a kind of accuracy are used in most scientific works [7, 24].

The measurements that had been obtained in this analysis were presented in absolute value of the angle, ignoring the underestimation or overestimation of the position (positive and negatives values). This was necessary for statistical calculations.

The ERJP results in the testing material reflect the capacity of healthy glenohumeral joints’ proprioception. The study group can be regarded as a control group for the comparison of JPS with other groups with potential proprioception deficits. In subsequent works of our team, the result of studies on patients with instability and rotator cuff injuries will be presented, in addition with the information about the capacities of proprioception in throwing sports. We have obtained an average error of reconstruction of a value from 3° to 6.1° with compliance to the terms of methodology and use of accurate devices. These results are similar to the data from other works of related topics. The biggest errors had occurred at lower positions of deviation and in movements of flexion and abduction. For abduction, flexion and internal rotation movements, the differences proved to be statistically significant (Table 3). The volume of work does not allow for a more detailed analysis, being the effect of the dominant hand or sex. This kind of scientific analysis is the subject of a separate work presented for publication.

**Table 3:** Statistical analysis by ANOVA; n.s.- nonsignificant

Reference position	*P* level (ANOVA)	
	Flexion	Abduction
60 vs. 90	0.05	<0.0001
60 vs. 120	0.05	<0.0001
90 vs. 120	n.s.	n.s.

Reference position	External rotation	Internal rotation
30 vs. 45	n.s.	0.03
30 vs. 60	n.s.	0.003
45 vs. 60	n.s.	n.s.

There are some limitations of the study. Ultimate evaluation of the method should be based on validation. That process in the way and will be presented separately. Also the results are limited for now and do not describe fully the normal shoulder proprioception. This issue will be the subject on another analysis.

## Conclusions

The designed device has created a possibility to perform precise measurements of the shoulder joints’ position. It is completely safe to use.

The evaluation of proprioception, expressed as sensory testing of the joint position, requires precise compliance with research protocol. Results obtained in the group of normal glenohumeral joints serve as reference for the study of proprioception in shoulder injuries, or the impact of treatment and training, on the joint position sense.
